# Perceived stress and gastrointestinal symptoms in nursing students in Korea: A cross-sectional survey

**DOI:** 10.1186/1472-6955-10-22

**Published:** 2011-11-08

**Authors:** Eun Young Lee, Mi Suk Mun, Seon Hye Lee, Ho Soon Michelle Cho

**Affiliations:** 1 Institute for Community Health, Hanyang University, Seoul, Korea; 2 Department of Nursing, Chungju National University, Jeungpyeong, Korea; 3 Department of Nursing, Dong U College, Sokcho, Korea; 4 College of Nursing, Texas Woman's University, Dallas, Texas, USA

## Abstract

**Background:**

Although nursing students experience a high level of stress during their training, there has been limited research on stress and its impact on the student's physical responses, such as gastrointestinal symptoms. The aims of this study are to assess the prevalence of GI symptoms in nursing students in Korea and to examine the association between the perceived stress and GI symptoms.

**Methods:**

A cross-sectional descriptive study design was used. A total of 715 students of a three-year associate degree nursing program in a Korean college participated. The Perceived Stress Scale and a GI Symptoms Questionnaire were administered through a self-reported system. Chi-square tests, Fisher's exact test, and logistic regression analysis were performed using SPSS 17.0.

**Results:**

Sixty-five percent of the nursing students experienced more than one GI symptom, with 31.1% of students reporting more than three GI symptoms. Most of the nursing students complained of upper dysmotility and bowel symptoms. In addition, students who reported higher perceived stress were significantly more likely to complain of GI symptoms. Compared to nursing students with the lowest perceived stress level, the adjusted odds ratio (OR) for GI symptoms in students with the highest perceived stress level was 3.52 times higher (95% CI = 2.05-6.06).

**Conclusions:**

GI symptoms that are highly prevalent among nursing students are significantly associated with the perceived stress level. High perceived stress should be considered a risk factor for GI symptoms. To reduce perceived stress, stress management programs including cognitive reappraisal training are needed in nursing curriculum.

## Background

Nursing students experience a high level of stress during their training. This stress comes not only from academics, but also from clinical stress arising from the theory-practice gap, along with possible poor relationships with the clinical staff [1-3]. Such stressors could lead to a negative coping style [4], burnout, and psychological morbidity [2,5]. There have been many studies on the level of stress, the sources of stress, and its impact on psychological response among nursing students. However, there has been limited research on stress and its impact on the student's physical responses, such as gastrointestinal symptoms.

Gastrointestinal (GI) symptoms, common in the world-wide population, create not only higher health care utilization, but also negatively impact daily activities and quality of life [6-8]. The vast majority of GI symptoms are associated with functional GI disorders, specific symptoms with no identifiable explanation [9,10]. Prevalence rates of GI symptoms ranging between 35% and 70% have been reported in several epidemiological surveys for other countries [11-13]. Norton et al. reported that GI symptoms were diagnosed in 51.2% of the 127 university students in Canada, and functional dyspepsia (22.8%), dyschezia (20.5%), and functional heartburn (19.7%) were the most frequently diagnosed disorders [14]. According to a survey for students of Zurich-based universities, about 64.2% reported at least one GI symptom; functional abdominal bloating (39.5%) and unspecified functional esophageal disorder (14.5%) were the most frequently reported disorders [15]. Although nursing students frequently complain of GI symptoms and are often absent from class due to these complaints, little is known about the prevalence of GI symptoms in nursing students.

The etiology of GI symptoms is unknown. However, George Engel's biopsychosocial model has provided a framework for understanding GI symptoms [16,17]. Engel argued that, since humans are biological, psychological and social beings, difficulty in any of these aspects can negatively affect all of them. Thus, there are many interrelated factors to cause disease [18]. Based on this model, the mechanisms that affect the relationship between stress and GI symptoms include, but are not limited to, the effects on gastrointestinal motility mediated by a visceral nervous system, the immune system, and the hypothalamic-pituitary-adrenal axis. A lowered threshold for perceiving adverse visceral sensations is also a factor [16,18]. However, there has been limited research on the relationship between stress and GI symptoms except for irritable bowel syndrome (IBS) in healthy populations.

Although many studies have focused on stressful events and chronic stress, the intensity or the number of stressful events is insufficient to cause pathology and illness. Recently, perceived stress that appears threatening is considered to be the most predictive factor for GI symptoms [15,19]. The perception of threat arises when the demands imposed upon an individual are perceived to exceed his or her felt ability to cope with those demands, irrespective of the source and the intensity or the number of stressful events.

Therefore, this study was conducted to assess the prevalence of GI symptoms in Korean nursing students and to examine the association between perceived stress and GI symptoms in the healthy population.

## Methods

### Research Design

This study was designed as a cross-sectional descriptive study to assess the prevalence of GI symptoms in Korean nursing students and to examine the association between perceived stress and GI symptoms.

### Sample and Ethical Considerations

Seven hundred and fifteen students of a three-year associate degree nursing program in a Korean college anonymously reported their responses on the questionnaires. Since the Korean Council for University Education introduced nursing education accreditation in 1997, the curriculums of nursing programs in Korea have been standardized. Thus, the colleges of nursing are similar to other colleges with nursing students. The Research Review Committee of Jinju Health College approved this study prior to data collection. Students were informed that (a) their participation was voluntary, (b) they could refuse to answer any item, and (c) there would be no adverse consequences for refusal. Signed informed consent to participate was obtained from all subjects. Data were collected from students who had completed at least one semester in the nursing program. It was determined that a convenient time for students to answer survey questions would be immediately after taking their final exams. The response rate was 98.6%.

### Measures

The self-report questionnaire included general information, as well as the Perceived Stress Scale and the GI Symptoms Questionnaire. The English versions were translated into Korean by researchers and reviewed by a nursing professor and an English professor. A pilot study was then performed using three nursing students.

General information included gender, age, year of study, religion, time staying at school, living arrangements, smoking, alcohol consumption, subjective health status, and height and weight. Subjective health status was assessed by the question: "What is your health status?" Responses were recorded on a 3-point scale, ranging from poor to good. The body mass index (BMI) was calculated as weight (in kilograms) divided by height (in meters) squared. According to the World Health Organization (WHO) Asian Pacific classification, the normal BMI range is 18.5-22.9 kg/m^2 ^in adult Asians [20].

Perceived stress was assessed using the Perceived Stress Scale (PSS 10; 10 items) [21,22]. The PSS measures the degree to which situations in one's life over the past month are identified as stressful. Items are designed to assess how unpredictable, uncontrollable and overloaded respondents find their lives. The PSS was designed for use in community samples with at least a junior high school education. The questions in the PSS ask about feelings and thoughts during the previous month. In each case, responses were recorded on a 5-point Likert scale, ranging from never (0) to very often (4). PSS scores were obtained by reversing responses to the four positive items and then summing across all scale items. A higher score means a higher level of perceived stress. Reliability and validity testing of this instrument was done by Cohen and Williamson [22]. Cronbach's alpha in this sample was 0.75.

GI symptoms were assessed using the 16-item Gastrointestinal Symptoms Questionnaire [9]. The questionnaire has five symptom categories: esophageal symptoms, upper dysmotility symptoms, bowel symptoms, diarrhea, and constipation. The items focus on the frequency of GI symptoms over the past three months. Symptoms are based on ROME II criteria. Responses were recorded on a 5-point Likert scale, ranging from not at all (0) to very often (4). The response options of "often" and "very often" were used to identify a symptom as present. Reliability and validity testing of this questionnaire was done by Bytzer and colleagues [9]. Cronbach's alpha in this sample was .83.

### Data Analysis

For descriptive analysis, data were expressed as frequency and percentage. Perceived stress was categorized into four groups based on a quartile. The first quartile (Q1) represents the lowest perceived stress level, while the fourth quartile (Q4) represents the highest perceived stress level. Chi-square tests and Fisher's exact tests were conducted to compare the categorical variables between groups with and without GI symptom. Differences among the quartiles of perceived stress (Q1~Q4) were analyzed using Chi-square tests. To analyze the relationship between perceived stress and GI symptoms, logistic regression analyses were conducted. Initially, we estimated the crude association between perceived stress and GI symptoms. Multiple logistic regression analysis for GI symptoms was conducted adjusting for the variables, which showed a significant difference in Chi-square tests. For all tests, significance was determined at a 2 tailed p value ≤ 0.05. Statistics analyses employed the Statistical Package for Social Science (SPSS), version 17.0.

## Results

### General Characteristics

Sixty-eight males (9.6%) and 641 females (90.4%) ranging in age from 16 to 35 years (mean age 20.59 ± 2.54 years) participated in the study. Half of the participants reported having a religion in the following order: Buddhist, Protestant, Catholic and others. The hours that the participants said they stayed at school were 32.5% less than 9 hours, 46.3% 9-13 hours, and 21.2% over 13 hours. Living arrangement included 40.3% in a parent's house, 29.6% in a rental house, 23.7% in a dormitory, 3.4% with a relative, 1.7% in lodging, and 1.3% designated as other. The smoking rate was 3.8%, and 64.5% of the participants drank alcohol. Self-evaluation for health status was 45.2% Good, 48.5% Fair, and 6.3% Poor. A normal BMI was reported in 72.1% of participants (Table 1).

**Table 1:** General Characteristics


**Characteristics**		**N (%)**

Gender	Female	641(90.4)
	Male	68(9.6)
	Total	709(100.0)

Age	< 20	279(39.7)
	20-24	362(51.5)
	25 ≦	62(8.8)
	Total	703(100.0)

Year of study	1st	310(43.8)
	2nd	249(35.2)
	3rd	149(21.0)
	Total	708(100.0)

Religion	Yes	357(50.0)
	No	357(50.0)
	Total	714(100.0)

Time staying in school	< 9 hrs	230(32.5)
	9-12 hrs	327(46.3)
	≧ 13 hrs	150(21.2)
	Total	707(100.0)

Living with parent	Yes	288(40.3)
	No	426(59.7)
	Total	714(100.0)

Smoking	Yes	27(3.8)
	No	688(96.2)
	Total	715(100.0)

Alcohol consumption	Yes	457(64.5)
	No	252(35.5)
	Total	709(100.0)

Subjective Health status	Good	322(45.2)
	Fair	346(48.5)
	Poor	45(6.3)
	Total	715(100.0)

BMI	Underweight (BMI < 18.5)	120(18.0)
	Normal weight (18.5 ≦BMI < 23.0)	497(72.1)
	Overweight (23.0 ≦BMI < 25)	50(7.3)
	Obesity (25 ≦BMI)	18(2.6)
	Total	685(100.0)

### Prevalence of GI Symptoms

Nursing students reported postprandial fullness (91.3%), bloating (88.9%), abdominal pain (87.8%), and diarrhea/constipation (84.7%) over the past three months. Among the symptoms which were responded to as "often" and "very often", were postprandial fullness (27.7%), bloating (25.8%), abdominal pain (17.6%), and diarrhea/constipation (26.6%) (Table 2). A total of 431 (65%) of the nursing students reported more than one GI symptom. Among those, 206 (31%) students reported over three GI symptoms. Specifically, 311 (45.7%) students reported bowel symptoms, 292 (39.1%) students reported upper dysmotility symptoms, and 85 (12.0%) students reported esophageal symptoms (Table 3).

**Table 2:** Prevalence of Gastrointestinal Symptoms


	**Not at all**	**Sometimes** **Rarely**	**Very often** **Often**
	
	**N (%)**	**N (%)**	**N (%)**

Abdominal pain	87(12.2)	502(70.2)	126(17.6)
Esophageal symptoms			
Dysphagia	490(68.7)	205(28.8)	18(2.5)
Heartburn	247(34.8)	390(54.9)	73(10.3)
Upper dysmotility symptoms			
Early satiety	173(24.2)	466(65.3)	75(10.5)
Postprandial fullness	62(8.7)	452(63.6)	197(27.7)
Bloating	79(11.1)	447(63.0)	183(25.8)
Nausea	351(49.1)	324(45.3)	39(5.5)
Vomiting	474(66.5)	226(31.7)	13(1.8)
Bowel symptoms			
Diarrhea/Constipation	109(15.3)	413(58.1)	189(26.6)
> 3 bowels each day	381(53.5)	299(42.0)	32(4.5)
Loose or watery stools	121(17.0)	514(72.2)	77(10.8)
Urgency	184(26.0)	478(67.4)	47(6.6)
< 3 bowels each week	268(37.8)	323(45.6)	118(16.6)
Hard or lumpy stools	112(15.8)	497(69.9)	102(14.3)
Anal blockage	334(47.2)	336(47.5)	37(5.2)
Fecal incontinence	608(85.6)	99(13.9)	3(0.4)

**Table 3:** Number of Gastrointestinal Symptoms Reported


	**items**	**No symptoms**	**1-2 symptoms**	**≧3 symptoms**
		
		**N (%)**	**N (%)**	**N (%)**

GI symptoms	16	232(35.0)	225(33.9)	206(31.1)
Esophageal symptoms	2	623(88.0)	85(12.0)	-
Upper dysmotility symptoms	5	428(60.9)	219(31.1)	73(8.0)
Bowel symptoms	8	370(54.3)	232(34.1)	79(11.6)
Diarrhea symptoms	3	576(81.8)	123(17.5)	5(0.7)
Constipation symptoms	3	525(75.1)	161(23.0)	13(1.9)

All symptoms counted if reported to occur often or very often.

### General Characteristics, Perceived Stress and GI Symptoms

Approximately two-thirds of female students experienced more than one GI symptom over the past three months, but there was no significant gender difference (χ^2 ^= 4.27, p > 0.05). Nursing students who reported poor subjective health status were significantly more likely to experience GI symptoms (χ^2 ^= 47.28, p < 0.001). No group differences were noted for age, year of study, religion, time staying in school, living with parent, smoking, alcohol consumption, and BMI (Table 4). Except for subjective health status, there was no significant difference in the level of perceived stress. Nursing students with poor subjective health status reported higher levels of perceived stress (χ^2 ^= 76.92, p < 0.001) (Table 5). Thus, these results indicated that subjective health status had a relationship with GI symptoms and perceived stress.

**Table 4:** Prevalence of Gastrointestinal Symptoms by General Characteristics


**Characteristics**		**With GI symptoms**	**Without GI symptoms**	**χ^2^**	**p**
		**N (%)**	**N (%)**		

Gender	Female	392(66.1)	201(33.9)	4.27	0.05
	Male	34(53.1)	30(46.9)		

Age	< 20	165(63.5)	94(36.5)	0.63	0.73
	20-24	220(65.7)	115(34.3)		
	25 ≦	39(68.4)	18(31.6)		

Year of study	1st	182(63.9)	103(36.1)	1.03	0.60
	2nd	150(64.1)	84(35.9)		
	3rd	94(68.6)	43(31.4)		

Religion	Yes	207(64.1)	116(35.9)	0.21	0.68
	No	223(65.8)	116(34.2)		

Time staying in school	< 9 hrs	131(60.4)	86(39.6)	3.15	0.21
	9-12 hrs	201(66.8)	100(33.2)		
	13 hrs ≦	95(68.3)	44(31.7)		

Living with parent	Yes	168(62.0)	103(38.0)	1.77	0.19
	No	262(67.0)	129(33.0)		

Smoking	Yes	13(54.2)	11(45.8)	1.29	0.28
	No	418(65.4)	221(34.6)		

Alcohol consumption	Yes	269(62.7)	160(37.3)	2.85	0.10
	No	158(69.3)	70(30.7)		

Subjective health status	Good	152(51.5)	143(48.5)	47.28	< 0.001
	Fair	240(74.1)	84(25.9)		
	Poor	38(90.5)	4(9.5)		

BMI	Underweight (BMI < 18.5)	66(57.4)	49(42.6)	6.93	0.07
	Normal weight (18.5 ≦BMI < 23.0)	316(68.7)	144(31.3)		
	Overweight (23.0 ≦BMI < 25)	29(64.4)	16(35.6)		
	Obesity (25 ≦BMI)	7(50.0)	7(50.0)		

**Table 5:** Perceived Stress Level by General Characteristics


**Characteristics**		**Level of Perceived Stress**				**χ^2^**	**p**
		**Q1**	**Q2**	**Q3**	Q4*		
				
		**N (%)**	**N (%)**	**N (%)**	**N (%)**		

Gender	Female	183(29.2)	131(20.9)	169(27.0)	144(23.0)	2.18	0.54
	Male	24(35.8)	15(22.4)	17(25.4)	11(16.4)		

Age	< 20	91(33.3)	57(21.0)	69(25.5)	54(19.9)	6.05	0.42
	20-24	96(27.0)	71(20.0)	101(28.5)	87(24.5)		
	25 ≦	18(29.0)	17(27.4)	14(22.6)	13(21.0)		

Year of study	1st	100(33.1)	67(22.2)	75(24.8)	60(19.9)	6.37	0.38
	2nd	67(27.2)	47(19.1)	67(27.2)	65(26.4)		
	3rd	41(28.3)	32(22.1)	43(29.7)	29(20.0)		

Religion	Yes	101(29.1)	67(19.3)	95(27.4)	84(24.2)	2.27	0.52
	No	107(30.5)	80(22.8)	92(26.2)	72(20.5)		

Time staying in school	< 9 hrs	73(32.9)	42(18.9)	51(23.0)	56(25.2)	4.94	0.55
	9-12 hrs	89(27.7)	71(22.1)	94(29.3)	67(20.9)		
	13 hrs ≦	45(30.4)	31(20.9)	40(27.0)	32(21.6)		

Living with parent	Yes	80(28.2)	69(24.3)	73(25.7)	62(21.8)	3.35	0.34
	No	128(30.9)	77(18.6)	114(27.5)	95(22.9)		

Smoking	Yes	8(30.8)	4(15.4)	7(26.9)	7(26.9)	0.66	0.88
	No	200(29.7)	143(21.2)	180(26.7)	150(22.3)		

Alcohol consumption	Yes	136(30.4)	104(23.3)	115(25.7)	92(20.6)	4.93	0.18
	No	72(29.3)	42(17.1)	70(28.5)	62(25.2)		

Subjective health status	Good	129(41.0)	70(22.2)	78(24.8)	38(12.1)	76.92	< 0.001
	Fair	77(22.8)	67(19.9)	100(29.7)	93(27.6)		
	Poor	1(2.2)	9(20.0)	9(20.0)	26(57.8)		

BMI	Underweight (BMI < 18.5)	35(30.4)	24(20.9)	28(24.3)	28(24.3)	6.36	0.70
	Normal weight (18.5 ≦BMI < 23.0)	151(30.9)	101(20.7)	129(26.4)	108(22.1)		
	Overweight (23.0 ≦BMI < 25)	14(28.6)	15(30.6)	13(26.5)	7(14.3)		
	Obesity (25 ≦BMI)	3(17.6)	3(17.6)	5(29.4)	6(35.3)		

*Q4 is the highest level of perceived stress.

### Relationship between Perceived Stress and GI Symptoms

Nursing students, who reported higher perceived stress, were significantly more likely to experience GI symptoms (χ^2 ^= 43.26, p = 0.00). Those students (83%) in Q4 experienced more than one GI symptom over the past three months, while 50.3% of students in Q1 experienced GI symptoms. These trends are shown in five symptom subcategories (Table 6).

**Table 6:** Prevalence of Gastrointestinal Symptoms by Perceived Stress level


	**Level of Perceived Stress**				**χ^2^**	**p**
	**Q1**	**Q2**	**Q3**	Q4*		
			
	**N (%)**	**N (%)**	**N (%)**	**N (%)**		

**GI symptoms**	**96(22.6)**	**83(19.6)**	**121(28.5)**	**124(29.2)**	**43.26**	**0.00**
Esophageal symptoms	15(18.5)	13(16.0)	20(24.7)	33(40.7)	18.60	0.00
Upper dysmotility symptoms	52(19.2)	53(19.6)	77(28.4)	89(32.8)	37.86	0.00
Bowel symptoms	65(21.2)	59(19.2)	88(28.7)	95(30.9)	32.48	0.00
Diarrhea symptoms	27(21.6)	22(17.6)	35(28.0)	41(26.5)	11.46	0.01
Constipation symptoms	38(22.2)	35(20.5)	44(25.7)	54(31.6)	13.32	0.00

*Q4 is the highest level of perceived stress.

The logistic regression analysis was used to study associations between perceived stress and GI symptoms. Table 7 displays the results of the unadjusted model (model I). Compared to nursing students with Q1, which represents the lowest perceived stress level, Q2 increased the odds ratio (OR) for GI symptoms 1.61-fold (95% CI = 1.03-2.53), Q3 increased the risk 2.22-fold (95% CI = 1.45-3.40), and Q4, the highest perceived stress level, increased the risk 5.11-fold (95% CI = 3.04-8.61). The association between perceived stress and GI symptoms remained significant in analyses adjusted for subjective health status (model II). The subjective health status-adjusted odds for GI symptoms were 1.34 (0.84-2.13) for Q2, 1.84 (1.19-2.87) for Q3, and 3.52 (2.05-6.06) for Q4.

**Table 7:** The Odds Ratio for Gastrointestinal Symptoms, unadjusted (Model I) and adjusted (Model II)


**Variables**		**Model I**	**Model II**
		**Odds ratio (95% CI)**	**Odds ratio (95% CI)**

Perceived Stress			
	Q1	1.00	1.00
	Q2	1.61 (1.03-2.53)	1.34 (0.84-2.13)
	Q3	2.22 (1.45-3.40)	1.84 (1.19-2.87)
	Q4*	5.11 (3.04-8.61)	3.52 (2.05-6.06)
Subjective Health Status			
	Good		1.00
	Fair		2.21 (1.55-3.14)
	Poor		5.49 (1.86-16.19)

*Q4 is the highest level of perceived stress.

## Discussion

This study was conducted to assess the prevalence of GI symptoms in Korean students (n = 715) enrolled in an associate degree nursing program and to examine the association between perceived stress and GI symptoms, using a cross-sectional descriptive study design. The biopsychosocial model outlined by Engel served as the main framework guiding this study.

According to the results, 65% of nursing students experienced more than one GI symptom and 31.1% of students experienced more than three GI symptoms over the past three months. Students reported upper dysmotility symptoms (postprandial fullness, bloating, abdominal symptoms) and bowel symptoms (diarrhea/constipation). Those students who reported higher perceived stress were significantly more likely to complain of GI symptoms. Thus perceived stress was significantly associated with GI symptoms among nursing students.

The prevalence of GI symptoms in Korean nursing students is similar to other reports. According to studies from North American, the prevalence of GI symptoms in a population-based survey was 61.7% of 1,149 Canadians [13] and 69% of 5,430 U.S. householders [11]. In the Greek general urban population, 53% reported more than one GI symptom during the past week and 55% during the past 6 months [6]. In addition, the prevalence of GI symptoms in students was 51.2% of 127 Canadian university students [14] and 64.2% of 668 university students in Switzerland [15].

Nursing students reported that the most frequent GI symptoms were the upper dysmotility symptoms and the bowel symptoms. Previous studies reported that the upper dysmotility symptoms were the most common type of GI symptoms [14,23,24]. However, the bowel symptoms were more common in Asians, while the esophageal symptoms were more common in Europeans [6,7,25]. Possible explanations for the lower prevalence of esophageal symptoms include the facts that Asians generally have a lower fat diet, a lower BMI, and a lower gastric acid output than Westerners [8].

In the present study, we found an association between perceived stress and GI symptoms among Korean nursing students. The association remained significant in analyses adjusted for subjective health status. This finding agrees with a biopsychosocial model. Perceived stress caused by high academic and clinical demands threatens the homeostasis of students and can have both a short- and long-term influence on the function of the GI tract [16]. A European cross-national study reported that university students with a high level of psychosocial stress were more likely to report psychosomatic complaints (OR 2.32; CI 1.86-2.89), including neck ache/backache (OR 1.34; CI 1.08-1.66), as well as GI complaints (OR 1.39; CI 1.12-1.71) [26]. Another European study showed that GI symptoms were significantly predicted by increased levels of perceived chronic stress, dispositional stress reactivity, and use of maladaptive coping strategies [15]. A similar association was reported in Korean studies. Han and colleagues reported that a high level of stress was significantly associated with irritable bowel syndrome (IBS) among medical, dental, and nursing students [27]. In addition, IBS was significantly predicted by increased levels of psychological stress (OR 1.87; CI 1.18-2.99) among participants in a health screening program [28]. Thus, the significant association between perceived stress and GI symptoms among Korean nursing students was confirmed in this study. This conclusion can be considered supportive evidence for the biopsychosocial model.

According to the results, subjective health status had a negative association with perceived stress and GI symptoms. A study, using the Cross-National Student Health Survey in Germany, Bulgaria and Poland, reported that subjective health status for university students had a strong association with psychosomatic complaints as well as physical and psychological health [29]. Cross-sectional approaches do not allow conclusions about causations. Thus, future studies are needed to investigate the causation among subjective health status, perceived stress, and GI symptoms. On the other hand, subjective health status is a feasible way to assess health in nursing students because it has been shown to have high reliability, validity, and predictive power for a variety of illnesses and conditions [30].

The prevalence of GI symptoms was 53.1% in male students in this study. It was slightly lower than female students (66.1%). Although gender differences were reported among general university students [15,26], it has not been confirmed in medical, dental, and nursing students [27,31]. Therefore, future studies are needed to investigate the relationships among gender, perceived stress, and GI symptoms between nursing students and others types of students. On the other hand, male students' health has been ignored in a predominantly female discipline such as nursing. Thus, nursing educators should also be concerned about stress-related diseases in male nursing students. The prevalence and risk factors for male nursing students should be examined.

This study has several limitations. First, our study participants consisted of a convenience sampling that might influence the generalizations of our results to the nursing students in Korea. Second, the design of this study was cross-sectional, which does not allow for a causal interpretation of our results. Third, a potential limitation of our study is the use of self-reporting measures for GI symptoms, although high correlations between diagnoses from questionnaires and from physical examinations have been reported [9]. Fourth, this study did not take into account other potential determinants of GI symptoms such as the use of non-steroidal anti-inflammatory drugs (NSAIDs). Fifth, data collection immediately after taking a final exam might influence one's perceived stress level and prevalence of GI symptoms, although results from this study are in line with several other reports [14,15,26-28].

Despite these limitations this study has several important strengths. To our knowledge, no previous study has assessed the prevalence of GI symptoms in nursing students. This study identifies the prevalence of GI symptoms and frequency of reported GI symptoms among Korean nursing students. In addition, this study revealed the association between perceived stress and GI symptoms in nursing students.

Based on the results, we suggest including stress management programs in formal and informal curriculum for nursing students. According to a systematic review for effectiveness of a stress management program in student nurses, the most effective interventions included skills for coping with stressful situations such as cognitive reappraisal and relaxation [32]. Thus, a stress management program focused on cognitive reappraisal training and physical and psychological skills for coping will contribute to the reduction of GI symptoms in nursing students.

## Conclusion

GI symptoms are frequent among Korean nursing students, similar to the reported prevalence in students in Western countries. Upper dysmotility and bowel symptoms are reported more frequently than esophageal symptoms. There is a significant association between GI symptoms and the student's perceived stress level. High perceived stress should be considered as a risk factor for GI symptoms. This information could be used to develop effective interventions to reduce the stress that students encounter. The results provide valuable information for other countries' educators in identifying students' stress and its effect on physical responses.

## Competing interests

The authors declare that they have no competing interests.

## Authors' contributions

EYL: Study design, data collection, data analysis, writing the manuscript. MSM, SHL: Study design, data analysis, writing the manuscript. HSMC: Literature review, writing and editing the manuscript. All authors read and approved the final manuscript.

## Pre-publication history

The pre-publication history for this paper can be accessed here:

http://www.biomedcentral.com/1472-6955/10/22/prepub
